# Author Correction: The impact of environmental variables on the spread of COVID-19 in the Republic of Korea

**DOI:** 10.1038/s41598-021-91674-6

**Published:** 2021-06-08

**Authors:** Yong Kwan Lim, Oh Joo Kweon, Hye Ryoun Kim, Tae‑Hyoung Kim, Mi‑Kyung Lee

**Affiliations:** 1 Department of Laboratory Medicine, Chung-Ang University College of Medicine, 102 Heukseok‑ro, Dongjak‑gu, Seoul, 06973 Republic of Korea; 2 Department of Urology, Chung-Ang University College of Medicine, Seoul, Republic of Korea

Correction to: *Scientific Reports* 10.1038/s41598-021-85493-y, published online 16 March 2021

In the original version of this Article, Hye Ryoun Kim was incorrectly listed as a corresponding author. The correct corresponding author for this Article is Mi-Kyung Lee. Correspondence and request for materials should be addressed to cpworld@cau.ac.kr.

In addition, Hye Ryoun Kim was incorrectly affiliated with ‘Department of Laboratory Medicine, Chung-Ang University College of Medicine, Heukseok-ro 102, Dongjak-gu, Seoul 156–755, Republic of Korea’. The correct affiliation is listed below.

Department of Laboratory Medicine, Chung-Ang University College of Medicine, 102 Heukseok-ro, Dongjak-gu, Seoul 06973, Republic of Korea.

The original Article has been corrected.

